# Association Between Alcohol Consumption and Psoriasis: Exploratory Analysis of Crowdsourced Web Search Data in Sweden

**DOI:** 10.2196/71992

**Published:** 2025-08-27

**Authors:** Anna Katharina Schober, Klas Nordlind, Alexander Zink

**Affiliations:** 1 Department of Medicine Solna Division of Dermatology and Venereology Karolinska Institutet Stockholm Sweden; 2 Department of Dermatology and Allergy Technical University of Munich Munich Germany

**Keywords:** addiction, alcohol, crowdsourcing, psoriasis, skin disease, epidemiology, infodemiology, alcohol abuse, web search data, internet

## Abstract

**Background:**

The nature of the relationship between psoriasis and alcohol consumption has been the topic of discussion for many years. Some studies have found that a higher intake of alcohol may be associated with a more severe manifestation of the disease. At the same time, patients with psoriasis often demonstrate elevated levels of alcohol consumption. It has not yet been fully established whether alcohol abuse serves as a trigger for psoriasis or if patients with psoriasis are simultaneously more prone to alcohol abuse.

**Objective:**

The objective of this study was to employ Google Trends as a tool for crowdsourcing data on a national level to explore the relationship between psoriasis and the consumption of alcohol in Sweden.

**Methods:**

This study examines crowdsourced web search data related to psoriasis and other skin disease–related search terms (such as utslag [rash]) as well as search interest in different types of alcohol. The analysis covers a 5-year period from 2018 to 2023 in Sweden, focusing on search behavior and correlations across this period.

**Results:**

The search behavior regarding psoriasis and alcohol-related search terms showed seasonal variations throughout the year. The relative search volume for psoriasis peaked in February, while alcohol-related searches, particularly Systembolaget and vodka, peaked in December and June. Our statistical analysis revealed relationships between the search interest regarding psoriasis and terms related to alcohol consumption, with disparities between different types of alcohol. The term “psoriasis” was negatively correlated with “Systembolaget” (*r*=–0.210), “vitt vin” (*r*=–0.224), and “vodka” (*r*=–0.220) (all *P*<.001), while the term “utslag” showed positive correlations with these same alcohol-related terms (*r*=0.278-0.347; *P*<.001).

**Conclusions:**

Crowdsourced data can offer valuable insights into population-level behavior. The observed negative correlations between psoriasis and alcohol-related searches suggest complex interactions, possibly reflecting reduced disease awareness or care during periods of higher alcohol consumption. The direction and strength of the correlations with psoriasis were not consistent across the different types of alcohol investigated in this study, which poses the question whether the relationship might be influenced by the type of beverage consumed. Further research is warranted to explore underlying mechanisms and validate these findings in clinical populations.

## Introduction

Around 3% of Europeans experience psoriasis, a chronic inflammatory skin disease characterized by papulosquamous plaques, often accompanied by itching [1]. As psoriasis frequently has a significant negative impact on patients’ quality of life and may lead to stigmatization and psychosocial impairment, mental illnesses represent a concern among such patients [2,3], and they tend to have relatively highly addictive behaviors, especially toward alcohol [4]. The relationship between psoriasis and alcohol consumption has been discussed for many years. Higher intake of alcohol may be associated with a more severe manifestation of the disease, which is unusual compared to other skin diseases [4]. Concurrently, patients with psoriasis often demonstrate elevated levels of alcohol consumption [4,5].

To approximate disease patterns and health care–seeking behaviors in the general population, crowdsourced data such as web search data have increasingly been employed in chronic and acute skin diseases for various approaches, including disease monitoring and the assessment of unmet needs and national disease [6,7]. With the data being publicly available and derived from the information-seeking behavior of large proportions of the general population [7], crowdsourcing represents a powerful tool in public health research.

In Sweden, the exclusive right to sell alcoholic beverages stronger than 3.5% alcohol by volume is held by Systembolaget—Sweden’s government-owned chain of liquor stores, which holds the state monopoly [8]. Thus, engagement with Systembolaget represents a unique opportunity to capture drinking behaviors at a population level.

This study aims to use web data related to skin diseases and alcohol consumption to further examine the interplay between psoriasis and alcohol in Sweden by crowdsourcing web search data at a national level.

## Methods

Google Trends was accessed in Sweden to analyze the public interest in search terms related to skin diseases and alcohol. This tool provides a relative search volume (RSV) metric, which quantifies the search interest for a given input within a defined region and timespan. RSV values range from 0 to 100, with 100 indicating the highest search volume during the specified period. The user input may be recognized as terms or topic, with terms referring to the exact words entered. Search topics are broader categories that Google identifies when it detects related phrases or concepts associated with a popular query. The latter can also be compared across different countries, as its identification is irrespective of language. We used only search terms, as they offer more transparency and control over the specific query content included in the analysis. Since this study was based on publicly available terms, institutional review board approval was not needed, and informed consent was not applicable.

The data collection spanned a 5-year period from June 2018 to June 2023 in Sweden. In this study, RSV data were collected for the search terms “psoriasis” and eczematous skin diseases (neurodermatit [atopic dermatitis], eksem [eczema], and utslag [rash]) as control. Neurodermatit and eksem represent the most common chronic inflammatory skin disease aside from psoriasis [9], providing a clinically relevant comparator. In contrast, utslag serves as a generic umbrella term encompassing acute and chronic skin eruptions of diverse etiologies, thus functioning as a broader population-level reference point. To capture general interest in alcohol purchase rather than preference for a specific beverage, the search term Systembolaget was included. Additionally, the selection of specific alcohol-related search terms was included to act as a proxy for patterns of alcohol interest, and thus, indirectly, consumption. These were vitt vin (white wine), rött vin (red wine), öl (beer), and vodka, chosen to represent both common beverage categories and a mix of lower-alcohol and higher-alcohol content drinks. All included search terms are listed in Table 1.

The statistical analysis encompassed the Wilcoxon and Kruskal-Wallis tests as well as Spearman correlations to examine the relationship between the search volumes of the selected terms. The analysis was conducted using the R software (version 4.3.0; R Foundation for Statistical Computing).

**Table 1:** Search terms.

	Minimum monthly RSV^a^	Maximum monthly RSV	Range
Utslag (rash)	56.3	83.5	27.2
Vitt vin (white wine)	25.8	54	28.2
Vodka (vodka)	29.5	62.5	33
Psoriasis (psoriasis)	36.3	69.8	33.5
Rött vin (red wine)	26.4	61	34.6
Öl (beer)	50.6	89.5	38.9
Eksem (eczema)	45.3	86.3	41
Systembolaget	19.3	65.5	46.2

^a^RSV: relative search volume.

## Results

RSV examination for the selected terms revealed noteworthy variations in search trends, both within and between years over the observed 5-year period. Notably, the yearly interest in the search terms psoriasis, eksem, utslag, Systembolaget, vitt vin, and vodka exhibited inconsistency. Statistical analysis confirmed significant differences in yearly RSVs among the 4 complete years included in the study (2019-2022) for all terms (*P*<.05). Similarly, the comparison of mean volumes by month yielded significant differences (*P*<.001) in mean RSVs throughout the year (Table 1). Specifically, the interest in psoriasis reached its zenith in February, followed by March and April. Likewise, the peak interest in eczema occurred in February, while the highest number of queries for rash was recorded in June, trailed by July and May. Regarding alcohol-related terms, the highest RSV was observed for Systembolaget and vodka in December succeeded by vitt vin in June or July. Notably, the interest in rash (utslag) exhibited the smallest overall range, with RSVs ranging from 56.3 to 83.5, while the interest in Systembolaget displayed the highest range, with RSVs ranging from 19.3 to 65.5.

**Figure 1:**
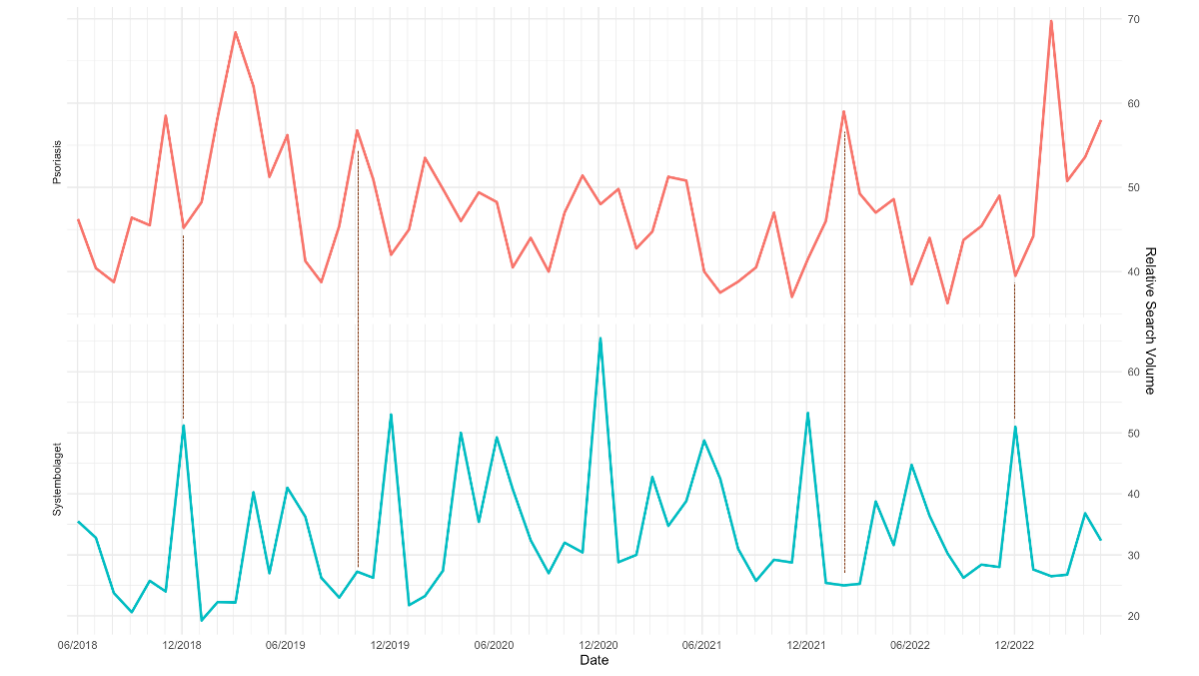
Relative search volume for Systembolaget and psoriasis (June 2018 to June 2023). Dark red dotted lines indicate selected time points where opposing patterns in search interest are most apparent, serving as a visual guide to illustrate periods of inverse search behavior.

Several associations were revealed in the correlation analysis. Negative correlations were obtained between psoriasis and Systembolaget (*r*=–0.21; *P=*.001; Figure 1), vitt vin (*r*=–0.22; *P*<.001), and vodka (*r*=–0.22; *P*<.001); the correlations with beer and red wine were nonsignificant (*r*<–0.1; *P*>.05).

In contrast, utslag was positively correlated with alcohol-related search terms such as Systembolaget (*r*=0.28), vitt vin (*r*=0.33), and vodka (*r*=0.35). These correlations were highly significant (*P*<.001). For eksem and neurodermatit, there was no evidence for correlations with the alcohol-related terms (*r*<0.1; *P*>.05).

## Discussion

RSV analysis for alcohol-related and skin disease–related search terms in Sweden through crowdsourcing provides a unique perspective into the relationship between alcohol and psoriasis on a population-based level. The differences in RSVs between years and seasons underscore the dynamic nature of public interest in these topics [10]. The distinct seasonal variations in interest, as evidenced by the peak months for psoriasis, eczema, rash, and alcohol-related terms, might indicate potential links between these factors and specific periods of the year [11,12].

The negative correlations between psoriasis and alcohol-related terms such as Systembolaget, vitt vin, and vodka raise questions about the relationship between alcohol consumption and the development or exacerbation of psoriasis. One possible explanation may be the temporary neglect of the disease during elevated alcohol intake, which may lead to impaired adherence to therapy or promote the development of long-term drinking habits [13-15]. Conversely, an individual with an existing addiction might not be affected by the seasonal variability in drinking habits. Other seasonal or environmental factors should also be considered, such as weather, which may influence both alcohol consumption and psoriasis severity independently, potentially confounding the observed associations. For example, reduced sunlight exposure in winter exacerbates psoriasis [16], which may contribute more directly to the observed peaks in search volume than behavioral changes alone. Other contributors to disease severity, such as dietary triggers or stress, could not be captured but may shape public interest over time.

Interestingly, the direction and strength of the correlations were not consistent across different types of alcohol. Regarding the effects of different alcohol types, the *Nordic Quality of Life Study* [17] found a negative correlation between psoriasis and the consumption of wine but not beer. To our knowledge, other than that, there is limited evidence on the effect of different types of alcoholic beverages on psoriasis, as research has focused on total alcohol consumption [4].

However, these correlations must be interpreted cautiously, as they do not establish a causal relationship between psoriasis and alcohol consumption. The underlying mechanisms and potential confounding factors that may contribute to these associations must be elucidated. Additionally, our reliance on web search data introduces inherent limitations such as the inability to ascertain the specific motivations and demographics of the individuals conducting the searches. Search behavior may be influenced by external factors such as media coverage, public health campaigns, or temporary events, which are difficult to control and may introduce bias. Further, there may be temporal or geographical inaccuracies in the data aggregation, which can affect the stability of the results. Due to the relative scaling of search volumes in Google Trends, direct comparisons between different search terms are less straightforward. Accordingly, we have limited cross-term comparisons and focused primarily on within-term trends such as seasonal patterns and the range of RSVs over time to ensure cautious interpretation.

Despite this, we underscore the value of crowdsourced web search data as a tool for investigating public interest and potentially identifying trends and patterns related to health conditions and behaviors. This approach opens new avenues for exploring the connection between psoriasis and alcohol consumption, complementing traditional research methods.

In conclusion, the analysis of search trends in Sweden provides intriguing insights into the relationship between psoriasis and alcohol consumption. The observed correlations between search volumes for alcohol-related terms and psoriasis-related terms warrant further investigation to enhance our understanding of the potential links between these factors. Future studies employing a multidimensional approach, including clinical data and patient-reported outcomes, can provide a more comprehensive understanding of the complex interplay between different alcohol sorts, their consumption, psoriasis, and associated inflammatory processes.

## Abbreviations

RSV: relative search volume

## Data Availability

The websearch data used in this study are publicly available on the Google Trends website [18]. The processed datasets generated during the current study are available from the corresponding author on reasonable request.
